# Is aggregated surveillance data a reliable method for constructing tuberculosis care cascades? A secondary data analysis from Uganda

**DOI:** 10.1371/journal.pgph.0000716

**Published:** 2022-11-23

**Authors:** Elizabeth B. White, Raúl U. Hernández-Ramírez, Robert Kaos Majwala, Talemwa Nalugwa, Tania Reza, Adithya Cattamanchi, Achilles Katamba, J. Lucian Davis

**Affiliations:** 1 Department of Epidemiology of Microbial Diseases, Yale School of Public Health, New Haven, CT, United States of America; 2 Uganda Tuberculosis Implementation Research Consortium, Makerere University, Kampala, Uganda; 3 Center for Interdisciplinary Research on AIDS, Yale School of Public Health, New Haven, CT, United States of America; 4 Center for Methods in Implementation and Prevention Science, Department of Biostatistics, Yale School of Public Health, New Haven, CT, United States of America; 5 National Tuberculosis and Leprosy Program, Ministry of Health, Kampala, Uganda; 6 Division of Pulmonary and Critical Care Medicine and Center for Tuberculosis, University of California, San Francisco, San Francisco, CA, United States of America; 7 Department of Medicine, Makerere University College of Health Sciences, Kampala, Uganda; 8 Pulmonary, Critical Care and Sleep Medicine Section, Yale School of Medicine, New Haven, CT, United States of America; Oxford University Clinical Research Unit Nepal, Patan Academy of Health Sciences, NEPAL

## Abstract

To accelerate tuberculosis (TB) control and elimination, reliable data is needed to improve the quality of TB care. We assessed agreement between a surveillance dataset routinely collected for Uganda’s national TB program and a high-fidelity dataset collected from the same source documents for a research study from 32 health facilities in 2017 and 2019 for six measurements: 1) Smear-positive and 2) GeneXpert-positive diagnoses, 3) bacteriologically confirmed and 4) clinically diagnosed treatment initiations, and the number of people initiating TB treatment who were also 5) living with HIV or 6) taking antiretroviral therapy. We measured agreement as the average difference between the two methods, expressed as the average ratio of the surveillance counts to the research data counts, its 95% limits of agreement (LOA), and the concordance correlation coefficient. We used linear mixed models to investigate whether agreement changed over time or was associated with facility characteristics. We found good overall agreement with some variation in the expected facility-level agreement for the number of smear positive diagnoses (average ratio [95% LOA]: 1.04 [0.38–2.82]; CCC: 0.78), bacteriologically confirmed treatment initiations (1.07 [0.67–1.70]; 0.82), and people living with HIV (1.11 [0.51–2.41]; 0.82). Agreement was poor for Xpert positives, with surveillance data undercounting relative to research data (0.45 [0.099–2.07]; 0.36). Although surveillance data overcounted relative to research data for clinically diagnosed treatment initiations (1.52 [0.71–3.26]) and number of people taking antiretroviral therapy (1.71 [0.71–4.12]), their agreement as assessed by CCC was not poor (0.82 and 0.62, respectively). Average agreement was similar across study years for all six measurements, but facility-level agreement varied from year to year and was not explained by facility characteristics. In conclusion, the agreement of TB surveillance data with high-fidelity research data was highly variable across measurements and facilities. To advance the use of routine TB data as a quality improvement tool, future research should elucidate and address reasons for variability in its quality.

## Introduction

Recent efforts to control and eliminate tuberculosis (TB), a leading infectious cause of death worldwide, have highlighted the importance not only of increasing access to diagnosis and treatment, but also of ensuring that TB care is of high quality [1]. Improving the quality of currently available TB services could avert up to one-third of all annual TB deaths, saving some 470,000 lives annually [2]. However, in many high-burden settings such as Uganda, these efforts are hindered by a lack of information about how and where to prioritize quality improvement (QI) efforts on local, regional, and national scales [3].

A widely respected framework advanced by Donabedian holds that the quality of health services depends on the structures, processes, and outcomes of care [4]. While measures of the structures of care are widely available [5], there is an ongoing need for better quality metrics that quantify the success of the key processes of accessing care, screening, diagnosis, and treatment that are required to achieve TB cure. These process and outcome metrics have been constructed through TB care cascades at the national level [6, 7] and among sub-populations [8–11] to identify where treatment gaps lie in different contexts. To date, these analyses have utilized study designs including modeling [7], systematic reviews with meta-analysis [6, 11], large population-based cross-sectional studies [8], and prospective cohorts [9, 10]. However, these approaches to constructing quality metrics are not able to be replicated in real time to inform QI efforts in many high-burden settings such as Uganda. An approach that utilizes the routinely collected, readily available, and representative data routinely collected by national TB programs would enable the use of quality metrics to inform program decisions.

In order to guide the use of routine TB data as a QI tool, it is important to understand how well it agrees with high-fidelity research data. A commonly cited disadvantage of routine data systems is that they are decentralized and without robust quality assurance systems, leading to variable data quality requiring adjustment (e.g. inventory and modeling studies to measure and correct undercounting of TB cases) [12–14]. Previous studies have taken various approaches to assess the quality of routinely collected health data with the ultimate goal of using it to inform program management, including examining the data’s consistency, timeliness, and completeness [15–17]. A more difficult dimension of data quality to measure is accuracy because of the lack of a gold standard. Validated methods are therefore needed to enable the use of surveillance data to produce timely, accurate measures of the quality of TB services [18, 19]. One promising approach, described by the WHO [20] and previously applied for maternal and newborn health, HIV care, acute respiratory infection, and immunizations in sub-Saharan Africa [21–23], is to evaluate the agreement of routine data with independently collected data.

In the current study, we used a similar approach to WHO by leveraging previously-collected source document data from a research study and comparing it to TB surveillance data from Uganda. Our objectives were to assess how well TB surveillance data agreed with high-fidelity research data collected from the same source documents, and to determine whether agreement changed over time, varied by health center, or was associated with facility characteristics. In doing so, we aimed to provide an example for researchers and public health leaders who wish to identify needs for future improvement in data capture toward the goal of producing real-time, routine data that can be used in QI efforts.

## Methods

### Study design and rationale

We conducted a secondary data analysis to assess agreement between two sources of routine TB data: 1) aggregated, facility-level surveillance data from the MOH’s national electronic disease reporting database (DHIS2, District Health Information Systems 2), and 2) individual patient data collected by the XPEL-TB research study. The XPEL-TB Study was a cluster-randomized trial examining the effect of a multicomponent diagnostic strategy on the number of TB patients initiating treatment within 14 days of diagnosis. The intervention included on-site GeneXpert testing for TB and monthly feedback of quality metrics to staff [24–26].

In the current study, both the surveillance and research datasets drew from the same source documents at the same health centers over the same time periods. Source documents included the TB treatment and laboratory registers, which are standardized logbooks used in Uganda and other LMIC settings to record all relevant TB laboratory testing and treatment data. A detailed description of how the surveillance and research datasets were collected from these registers is provided below in the *Data collection and reporting practices* section. We hypothesized that agreement between surveillance and research datasets might vary across measurements, health centers, and time; our study was designed to examine these dimensions to better understand the quality of TB data in this setting.

### Study setting and population

Our study was conducted in Uganda, one of WHO’s high HIV and TB burden countries [27], with an estimated TB prevalence of 253 per 100,000 population [28]. TB diagnostic and treatment services are overseen by the Ministry of Health’s (MOH’s) National Tuberculosis and Leprosy Programme (NTLP) and provided free of charge in primary health centers. Our study included 32 of these facilities from 18 urban, semi-urban, and rural districts in the Central and Eastern regions of Uganda. We included all XPEL TB study sites that participated in the baseline assessment and/or main trial (2017 and 2019); all sites were chosen because they had a high volume of smear examinations (>150) and smear-positive diagnoses (>15) per year [24]. Our analyses included data from January 1 through December 31, 2017 and January 1 through December 31, 2019; data for 2018 was not available for the research study.

The XPEL-TB study included all adults and children undergoing evaluation for possible TB, defined as having ≥1 sputum sent for smear or Xpert testing. For our analyses including surveillance data, we followed these inclusion criteria as closely as possible, as described below in “Measurements.”

### Data sources and recording practices

Surveillance data: In Uganda, all hospitals and health centers that treat TB patients report surveillance data on a quarterly basis following a series of steps. First, a trained staff member at each facility reviews handwritten TB laboratory and treatment registers quarterly and counts the number of new laboratory tests performed, laboratory test results, and case notifications. Second, the staff member records these aggregated counts on a standardized paper reporting form within pre-specified strata for case notifications (e.g., prior treatment status, disease type) and laboratory tests (smear microscopy, GeneXpert). Finally, either a facility-based or a district-level data officer reviews the reporting form and enters the data into a DHIS2 database. DHIS2 is a widely-used, open-source, health management information system that was implemented in Uganda in 2012, began collecting TB data in 2017, and was fully adopted for TB reporting in 2018 [29]. For this study, we extracted annual, facility-level data on diagnoses and treatment initiations from the quarterly reports in DHIS2.

High-fidelity research data: In the XPEL-TB study, trained facility staff photographed handwritten TB laboratory and treatment registers and uploaded them to a secure server monthly. Study staff then entered the data into a patient-level database and conduct quality assurance activities to ensure accuracy, including resolving missing data and other discrepancies with health facility staff [24]. The final dataset included patient results and dates for all steps of TB evaluation and treatment. To make direct comparisons to the surveillance dataset, we aggregated individual patient data by year and health center using the same strata reported in the DHIS2 system.

### Measurements

We selected a total of six measurements to compare between surveillance and research data. Four of these were TB care cascade measurements that were available in both the research dataset and quarterly surveillance reports [18]: smear-positive diagnoses (Smear positive), GeneXpert-positive diagnoses (Xpert positive), bacteriologically confirmed treatment initiations (BC treated), and clinically diagnosed treatment initiations (CD treated). BC treated included smear- and Xpert-positive patients who initiated TB treatment, while CD treated included those who were started on TB treatment without a confirmed diagnosis because they were deemed to have a high probability of pulmonary TB. We excluded the following from the BC and CD treated surveillance measurements: those previously treated for TB, those diagnosed with extra-pulmonary TB, those with evidence of drug-resistant TB, and those who transferred in from other health centers.

Finally, we included two measurements specific to people living with HIV (PLHIV). Among those initiating treatment for TB, we compared the number who were also PLHIV and the number who were taking antiretroviral therapy (ART) between the two data sources.

### Statistical analyses

All analyses were conducted using R version 4.1.2. First, we used descriptive statistics (median, interquartile range) and plots to summarize the distribution of data for each measurement in 2017 and 2019. We then used previously described methods [30–32] to calculate metrics of agreement and further characterize the relationship between surveillance and research data (described in Table 1).

**Table 1:** Agreement metrics, equations, and interpretations.

Statistic	Definition	Interpretation
Average Ratio	Average ratio of surveillance counts to research counts.	Overall agreement across health facilities. If <1, indicates underreporting in surveillance data relative to research data; if >1, indicates overreporting.
95% Limits of Agreement (LOA)	Upper and lower bounds for the average ratio within which 95% of ratios are expected to fall.	Expected range of agreement at the health facility level.
Concordance Correlation Coefficient (CCC)	Proportion of variation in counts attributable to facility, *assuming a fixed effect of data type*, ranging from 0 (none) to 1 (all).	Agreement was defined as high (CCC>0.75), moderate (0.50<CCC<0.75), or low (CCC<0.50).* The CCC is equivalent to the intraclass correlation coefficient (ICC) for agreement.

*We define high, moderate, and low agreement using the cutoffs for reliability commonly used in the literature for ICC [33].

For each of the six measurements, we calculated the average ratio of surveillance counts to research counts and the 95% Limits of Agreement (LOA) from the linear mixed model (Equation A in S1 Text) and the calculations (Table A in S1 Text) described in the Supplement using the R package “lme4.” The 95% LOA provide a wider range than 95% confidence intervals because they also incorporate the variance contributed by health center differences; the 95% LOA can be interpreted as the expected range of health center-level agreement. Next, we assessed agreement between the research and surveillance datasets by calculating Concordance Correlation Coefficients (CCCs). We used a variance components approach [30, 34], which calculates CCC directly from the variance components of linear mixed models that account for repeated facility-level measures over time. The linear mixed model was estimated using the “lme4” package in R; models (Equation B in S1 Text) and calculations (Table A in S1 Text) are further described in the Supplement. The CCC is equivalent to the Intraclass Correlation Coefficient (ICC) for agreement, measuring the proportion of variation in a measurement that is attributable to health facility differences adjusted for the effect of data type on the measurement; similar to ICC, the CCC ranges from 0 to 1 with 1 indicating perfect agreement between data types.

Second, we wanted to evaluate whether agreement between surveillance and research data changed over time, to 1) determine the suitability of surveillance data for monitoring performance trends and 2) identify possible changes in data quality after the full adoption of the DHIS2 system for TB reporting in 2018. To do this, we used the uncontrolled pre-post model described in Equation A with year (2017 vs. 2019) as a fixed effect. In addition, we used plots visually assess whether health facility-level agreement was consistent over time.

Finally, we sought to identify possible factors associated with agreement between research and surveillance data, including TB testing volume (measured as the total number of smear examinations), health facility level (subcounty or county), and location (Eastern or Central region). We included these characteristics as fixed effects in linear mixed models (see Equation C in S1 Text) predicting the magnitude of the difference between paired observations of surveillance and research counts in 2017 and 2019. Covariates were obtained from the surveillance database (DHIS2), the Ministry of Health’s master list of health facilities [35], and the Uganda Bureau of Statistics website [36].

### Ethical considerations

This study was determined to be exempt as not human subjects research by institutional review boards at Yale University and the Makerere University School of Public Health.

## Results

### Description of study sites and variables

**Fig 1:**
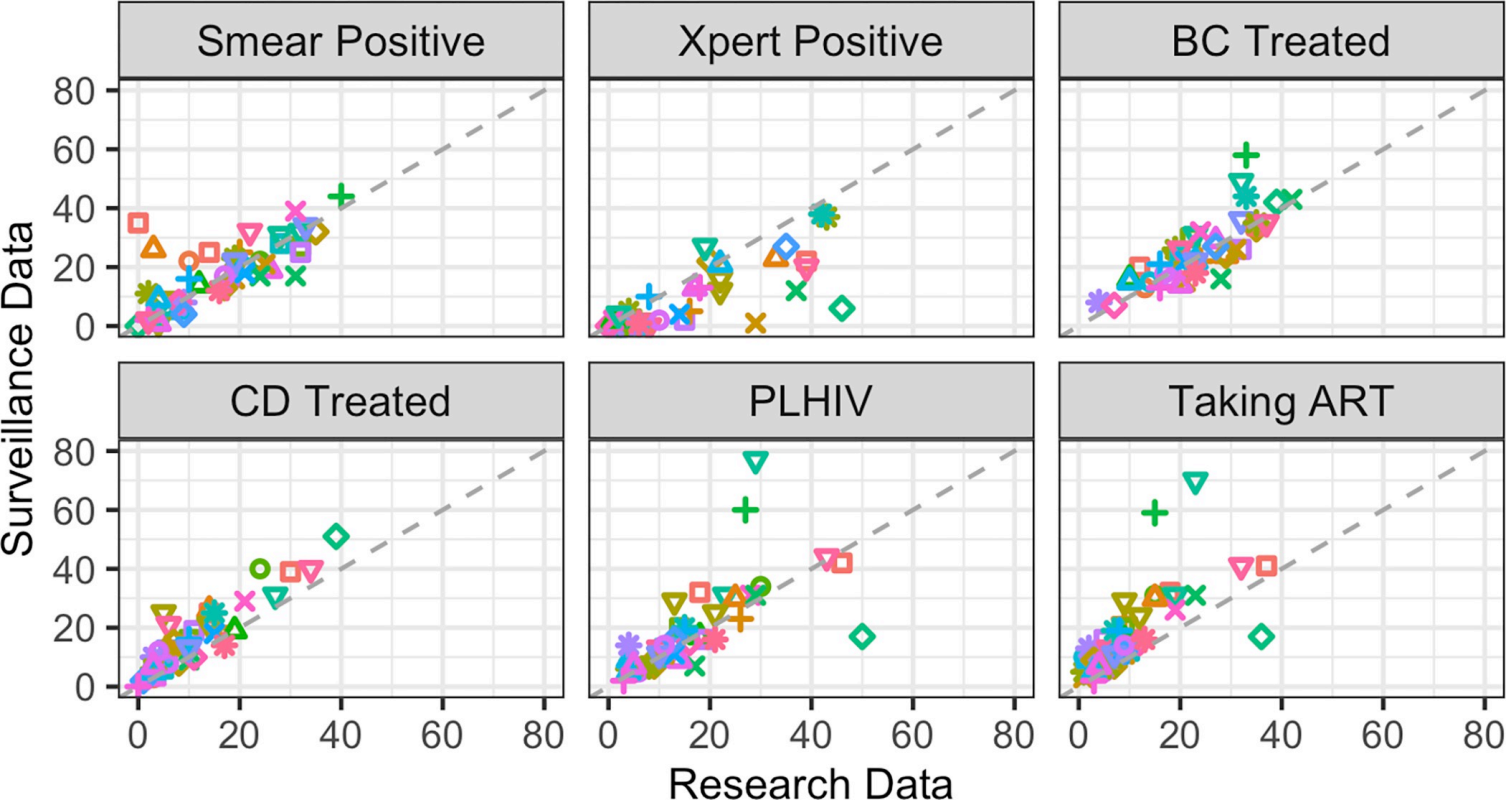
Scatterplots showing the relationship between research data (x-axis) and surveillance data (y-axis) counts for six measurements. Each of the 32 facilities is represented by a unique color and shape combination that is consistent across measurements. Two outliers with surveillance data counts >80 are not shown (both from Clinically Diagnosed and Treated (CD Treated) measurements).

The study sample included 32 government primary health centers, of which 24 were from the 2017 XPEL-TB baseline assessment, 20 were from the 2019 main trial, and 12 were included in both. 16 (50%) were Health Centre IIIs (subcounty-level) and 16 (50%) were Health Centre IVs (county-level), all located in 19 districts within 150 km of Kampala. According to the surveillance data for 2017 and 2019, these health centers had a median of 17.5 (IQR 9.0, 12.25) smear positive and 2.0 (IQR 0, 13.0) Xpert positive TB patients per year. A median of 24.0 (IQR 17.5, 31.25) bacteriologically confirmed TB patients and 14.0 (IQR 8.0, 25.0) clinically diagnosed TB patients initiated treatment at these sites. Of those who initiated treatment, a median of 14.5 (IQR 9.0, 26.0) were PLHIV, and a median of 14.0 (5.0, 24.5) were also taking ART (Table 2). Scatterplots in Fig 1 show the relationships between surveillance and research counts for each measurement; while all appear to be correlated, each measurement shows some variation from perfect agreement.

**Table 2:** Distribution and metrics of agreement comparing facility-level, annual surveillance data counts to research data counts for 2017 and 2019.

Measurement N*	Surveillance Data Median (IQR)	Research Data Median (IQR)	Avg. Ratio (95% LOA)	CCC
Smear Positive** (N = 768)	17 (9.0, 25.0)	18 (8.0, 25.5)	1.04 (0.38, 2.82)	0.783
Xpert Positive (N = 350)	2.0 (0, 13.0)	7.5 (3.0, 22.0)	0.45 (0.099, 2.07)	0.361
BC Treated (N = 1112)	24.0 (17.5, 31.25)	23.0 (18.75, 31.25)	1.07 (0.67, 1.70)	0.816
CD Treated (N = 882)	14.0 (8.0, 25.0)	10.0 (4.0, 15.25)	1.52 (0.71, 3.26)	0.822
PLHIV (N = 841)	14.5 (9.0, 26.0)	13.5 (9.0, 21.5)	1.11 (0.51, 2.41)	0.818
Taking ART (N = 808)	14.0 (5.0, 24.5)	9.0 (5.0, 13.5)	1.71 (0.71, 4.12)	0.616

**Abbreviations:** IQR = interquartile range, LOA = limits of agreement, CCC = concordance correlation coefficient, BC = bacteriologically confirmed, CD = clinically diagnosed, PLHIV = people living with HIV, ART = antiretroviral therapy.

*N is the total across all facilities and years, obtained from surveillance data

**one outlier excluded.

**Fig 2:**
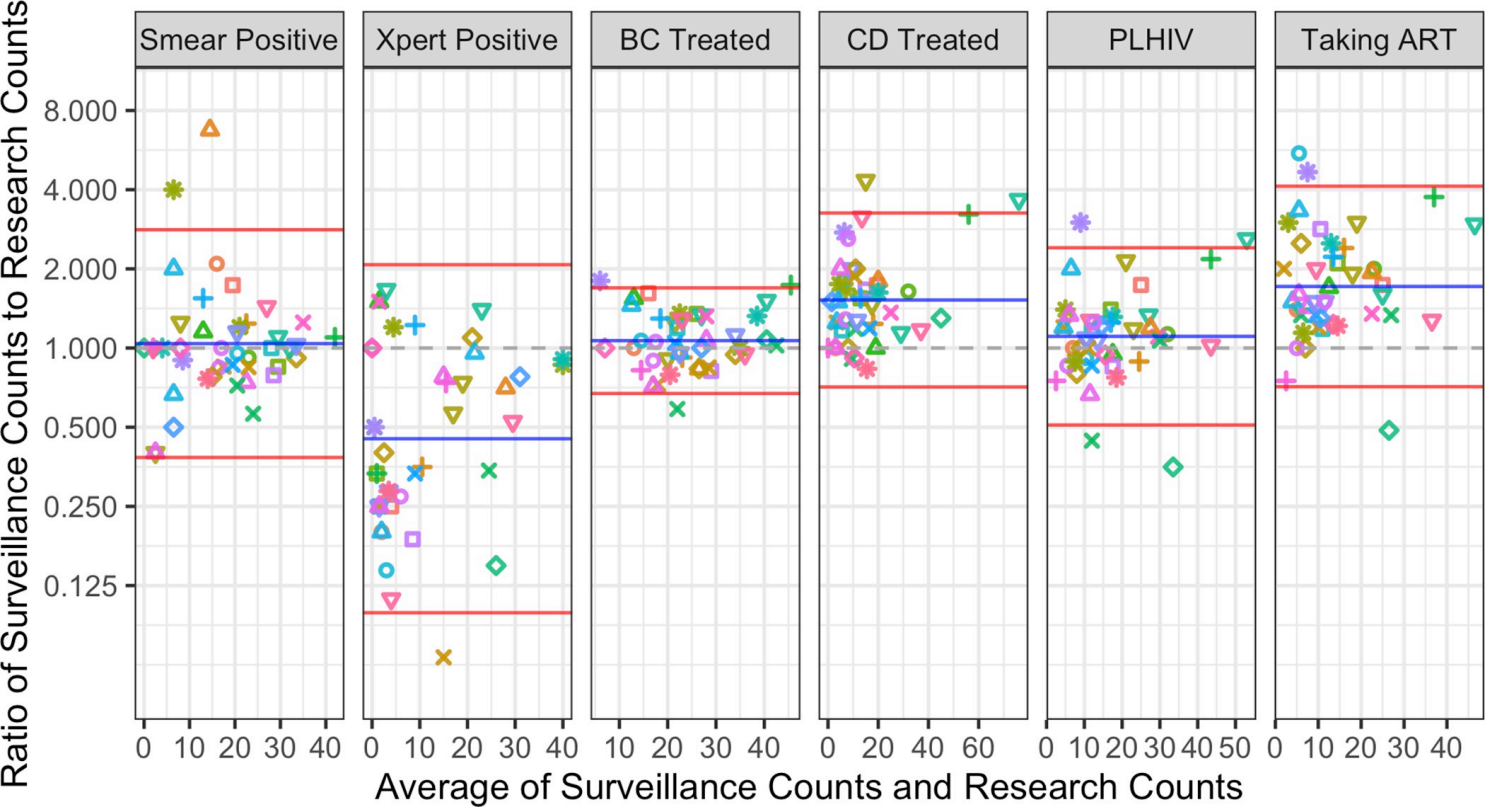
Bland-Altman plots showing the relationship between the average count and the ratio. For each measurement, the average of surveillance and research data counts (x-axis) versus the ratio of surveillance to research data counts (y-axis, log base 2 scale) in both 2017 and 2019 combined [37]. Gray dashed lines represent perfect agreement (ratio of 1), blue lines represent systematic agreement (average ratio), and red lines represent the expected range (95% LOA). Each facility is represented by a unique color and shape combination that is consistent across years and measurements.

For all six measurements, results of analyses assessing average agreement (ratio of surveillance counts to research counts), expected range of agreement (95% LOA), and agreement between data sources (CCC) are shown in Table 2 and Fig 2.

### Smear positive diagnoses

For the smear positive measurement, there was good agreement between surveillance and research data overall (average ratio = 1.04, CCC = 0.783). However, the 95% LOA revealed some facility-level variation; surveillance data measurements could be expected to be 62% lower to 180% higher than research data measurements.

### Xpert positive diagnoses

Xpert positive diagnoses showed poor agreement between surveillance and research data; they were counted on average 55% lower in the surveillance data with a wide range of expected facility-level agreement (mean ratio 0.45, 95% LOA 0.099–2.07). A low CCC below 0.500 indicated that between-data source differences, rather than between-facility differences, contributed much of the variation in Xpert positive measurements.

### Bacteriologically confirmed treatment initiations

BC treatment initiation counts showed good overall agreement between the two data sources (mean ratio = 1.07, CCC = 0.816). There was a moderate range in the expected agreement ratio at the facility level (95% LOA 0.67–1.70).

### Clinically diagnosed treatment initiations

For CD treatment initiations, surveillance data systematically overcounted relative to research data by an average of 52%; there was also a wide range of expected facility-level agreement (95% LOA 0.71–3.26). The CCC was high (CCC = 0.822), suggesting that most of the differences in this measurement were due to variation between health facilities; however, this measurement had particularly high variance contributed by both health facility and data source.

### People living with HIV

The number of people treated for TB who were PLHIV had good overall agreement between data sources with a wide range of facility-level agreement (average ratio = 1.11, 95% LOA = 0.51, 2.41) and a high CCC (0.818).

### People taking ART

The number of people treated for TB who were PLHIV taking ART was systematically overcounted in surveillance data relative to research data, with a wide range of expected facility-level agreement (average ratio = 1.71, 95% LOA = 0.71–4.12). A moderate CCC of 0.616 indicated that variance in this measurement came from both data sources and health facilities.

### Change over time and sources of variation

**Fig 3:**
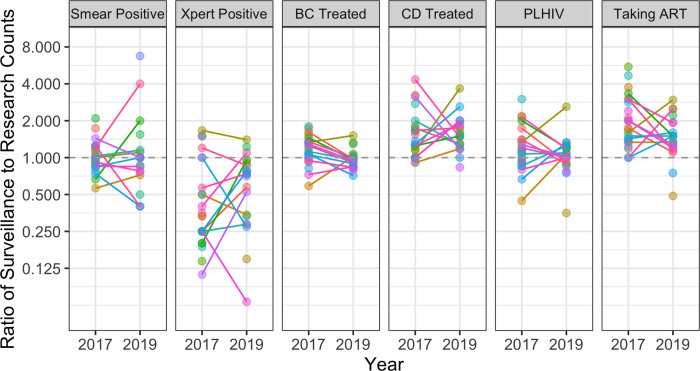
Change in facility-level agreement ratios between 2017 and 2019.

Comparing 2019 results to those from 2017, we saw that the systematic agreement between surveillance and research data did not differ substantially between the two years, in spite of concurrent programmatic efforts to improve the quality and usage of data through the DHI2 capture and reporting system. In the unadjusted pre-post analysis, year was not a statistically significant predictor of agreement for any measurements except BC Treated and Taking ART; even for these measurements, changes in point estimates for the average ratios from 2017 to 2019 were qualitatively small, remaining close to 1 for BC treated and between 1.45 and 2.0 for Taking ART (Table 3). However, there was evidence that small changes in average agreement might mask underlying fluctuations in facility-level agreement, as shown by plotting changes in facility-level agreement ratios between 2017 and 2019 (Fig 3). Results were similar when restricted to include only those facilities present in both the 2017 and 2019 datasets (Fig A in S1 Text).

**Table 3:** Results of analyses examining change in agreement between surveillance and research data over time: Metrics of agreement and pre-post analysis for 2017 (N = 24) and 2019 (N = 19).

Measurement	Average Ratio (95% LOA)		Effect of Year on Average Ratio (p-value)		CCC	
	2017	2019	2017	2019	2017	2019
Smear Positive	1.02 (0.25, 4.16)	1.05 (0.07, 17)	ref.	1.24 (0.29)	0.837	0.760
Xpert Positive	0.40 (0.05, 3.54)	0.52 (0.05, 5.22)	ref.	1.31 (0.23)	0.300	0.447
BC Treated	1.15 (0.31, 4.19)	0.97 (0.39, 2.44)	ref.	0.85 (0.02)*	0.801	0.848
CD Treated	1.51 (0.20, 11.2)	1.54 (0.09, 25.4)	ref.	1.02 (0.88)	0.714	0.869
PLHIV	1.16 (0.21, 6.32)	1.05 (0.13, 8.19)	ref.	0.90 (0.38)	0.749	0.878
Taking ART	1.97 (0.29, 13.5)	1.45 (0.19, 11.0)	ref.	0.73 (0.03)*	0.524	0.748

*significant at P<0.05 level

Finally, there were no significant associations between health facilities’ TB testing volume, level, or region with the magnitude of agreement between surveillance and research data (Table B in S1 Text).

## Discussion

As quality of care takes on greater priority in global TB control and elimination efforts, there is a need for timely and accurate data to monitor the quality of TB care and drive public health decision-making [2, 38]. The ready availability of surveillance data makes it attractive for this purpose, but it remains underused compared to research data. In this study from Uganda in 2017 and 2019, we found that these two types of data did not reliably agree, with variation occurring across measurements, between health facilities, and within facilities over time. Importantly, we saw that even variables with good systematic agreement overall, as measured by average ratios or CCCs, may have moderate to substantial underlying variability at the facility level. These findings suggest that data collection and reporting practices occurring at the facility level may be important drivers of data quality and, further, that systems should evaluate data quality in real time.

Understanding the sources of discrepancies in routine data is critical to correcting them, with the goal of ensuring high-quality data and enabling confidence in data use [39]. Previous research in multiple settings (e.g., TB inventory studies, which estimate TB incidence and reporting rates by linking patient records across multiple sources) has identified undercounting of health outcomes in routine data due to both low case ascertainment and under-reporting, and proposed methods for adjustment [12–14]. Our study was designed to evaluate underreporting among those patients who did seek TB care services. We expected to find that the surveillance data would either agree with or undercount diagnoses and treatment initiations compared to research data; however, we also found overreporting in some cases. Smear positive diagnoses and bacteriologically confirmed diagnoses had good overall agreement with research data. These two data elements have been the targets of NTLP quality assurance programs, are recorded in a centralized location on the original handwritten records, and represent the majority of TB patients in this setting [28]. Likewise, there are several possible explanations for the systematic undercounting of GeneXpert positive diagnoses in surveillance data relative to research data. In our study setting, delays in GeneXpert testing results, the lack of a dedicated register for this information, and the presence of multiple data collection tools are known challenges that could have contributed to under-recording of test results in the treatment registers [40]. However, we also found that clinically diagnosed TB treatment initiations were systematically higher in surveillance data compared to research data. While this was unexpected, it is similar to findings of overreporting of immunization data in quarterly reports compared to source documents, suggesting under-recording in the original source documents compared to other data tools [23, 41]. The quality assurance procedures in the research study may have led to the reclassification of some of these “clinically diagnosed” TB patients that appeared in the original handwritten records as bacteriologically confirmed.

For quality improvement programs, which aim to identify and act upon gaps in health system performance, some level of disagreement between surveillance and research data could be allowable if they are consistent over time and across settings. However, a major finding of this study was a great deal of underlying heterogeneity that could only partially be explained by health facility and time. Other studies in Uganda and Zimbabwe have found that staffing, supervision, and local use of data are associated with improved data quality [41, 42]. Although we did not identify statistically significant predictors of data agreement and the availability of these factors was limited by the secondary data analysis design, our approach proved feasible and could be adapted to other settings to identify factors associated with data quality. This approach also extends current WHO guidance [39] for assessing internal and external consistency of data over time by enabling the identification of factors associated with data consistency over time.

This study adds to a growing literature on the usefulness of routine data sources, such as public health surveillance data, for addressing gaps in the quality of TB care. However, most widely used methods, such as TB inventory studies and studies that generated national- and sub-national TB care cascades, have relied on routine data at the level of the individual patient, rather than the aggregated data that is available in many high-TB burden settings. In order for high-quality measurements to be available to program managers in real time in settings that collect only aggregated data, they need data that is reliable over time and accurately represents the care that is being provided. Strategies such as data audits with feedback to frontline health workers have been shown to improve both the quality of the recorded data and of the care provided [43]. Our study identifies some potential targets for these and other data quality improvement efforts, such as facilities with consistently poor reporting or data elements with higher inaccuracy. However, in order to fully address variability in the quality of routine data and increase confidence in its use, future research should seek to identify the underlying mechanisms for this variation. Qualitative and mixed-methods may be especially suited to answer these questions by characterizing the experiences and characteristics of those who record and compile routine data to inform efforts to improve the reliability of data collection [44].

Our study has several limitations. First, while we were very careful to match the study population and measurement definitions between the two data sources as closely as possible, it was not possible to achieve a perfect match for diagnoses, which are not stratified in the surveillance data to enable exclusion of patients believed to have a drug-resistant TB, who transferred from another facility, or who had previously been treated for TB in the past year. However, these groups represent a small minority of the patient population receiving TB care in Uganda: less than 10% of TB patients have been previously treated [45], and of that small proportion, an estimated 12% have evidence of drug resistance [46]. Thus, only 1% of new TB cases are likely previously treated with drug resistant TB, and unlikely to bias our results. Second, data on treatment outcomes was not available for the research study and data on possible TB patients was not available in the quarterly surveillance dataset; we were not able to examine these important measurements in our analysis. Third, this study used a convenience sample of twelve health centers in Central and Eastern Uganda; while this sample is representative of Uganda, further insights may be gained by assessing agreement between data sources in a larger sample, including additional countries. Finally, while our study benefitted from the availability of a research study that included comprehensive, high-fidelity data collection, data of this type is rarely available and alternative ways of collecting such data feasibly and at low-cost will be necessary to replicate and scale our approach.

Our study also has strengths. First and foremost, our approach could be broadly applicable in high TB burden settings as they continue to develop analytical approaches to monitor the quality of their surveillance data. We used an innovative approach to collect data from source documents as proof of principle for a reference standard against which to compare routinely reported data. This strategy could be incorporated into a quality assurance system, using a representative sample of data to estimate its accuracy without relying on comprehensive, prospective data collection or the availability of research data. One approach could be to use lot quality assurance sampling, which is already widely used for monitoring the performance of smear microscopy centers [47]. In addition, assessing agreement across locations, measurements, and time provides the opportunity to identify many different sources of variation in surveillance data, which may be highly specific to a particular context. Finally, our methods are suitable to be used with the aggregated count data available in many settings.

## Conclusions

Our study found substantial variability in the agreement of TB surveillance data with high-fidelity research data in a high-burden setting; agreement was best for smear positive diagnoses and bacteriologically confirmed treatment initiations and worse for Xpert positive diagnoses and clinically diagnosed treatment initiations. This study provides an example of how to use an analytical approach to identify sources of inaccuracy on the local scale and future studies should explore ways that it can be replicated on regional and national scales. Incorporating this approach as part of a regular quality assurance program would promote trust in routine TB data, ultimately enabling its use as part of a data-driven public health approach to ensuring high-quality care for people with TB.

## Supporting information

S1 Text (DOCX)
