# Supplementary material for: Is aggregated surveillance data a reliable method for constructing tuberculosis care cascades? A secondary data analysis from Uganda
Source: PLOS Glob Public Health. 2022 Nov 23;2(11):e0000716. doi: 10.1371/journal.pgph.0000716 (PMC10045605; doi:10.1371/journal.pgph.0000716)
Supplement: S1 Text — (DOCX) [file pgph.0000716.s001.docx]

**Supplementary Materials**

*Metrics of Agreement*

**Equation A.**

$$Y_{it}=\mu+\alpha_{i}+\gamma_{t}+e_{it}$$

$Y_{it}$= difference in log-transformed counts (surveillance data – research data)

$\mu$ = overall mean

$\alpha_{i}$ = random health facility effect

$\gamma_{t}$ = fixed year effect

$e_{it}$ = random error effect

**Equation B.**

$$Y_{ijt}=\mu+\alpha_{i}+\beta_{j}+\gamma_{t}+\alpha\beta_{ij}+\alpha\gamma_{it}+\beta\gamma_{jt}+e_{ijt}$$

$Y_{ijt}$= log-transformed count

$\mu$ = overall mean

$\alpha_{i}$ = random health facility effect

$\beta_{j}$ = fixed data source effect

$\gamma_{t}$ = fixed year effect

$\alpha\beta_{ij}$ = random health facility-data source interaction effect

$\alpha\gamma_{it}$ = random health facility-year interaction effect

$\beta\gamma_{jt}$ = fixed data source-year interaction effect

$e_{ijt}$ = random error effect

**Table A.** Formulas for metrics of agreement.^28^

| **Statistic** | **Equation** | **Calculations** | **Definitions** |
| --- | --- | --- | --- |
| Average ratio | S1 | $exp\left( \mu\right)$ | $\beta_{0}$: average difference between log-transformed surveillance and research counts  ${SD}_{total}$: total standard deviation  $\sigma_{\alpha}^{2}$: health facility variance  $\sigma_{\varepsilon}^{2}$: residual variance |
| 95% Limits of Agreement (LOA) | S1 | $exp\left( \mu\pm1.96*{SD}_{total} \right)$  ${SD}_{total}=\sqrt{\sigma_{\alpha}^{2}+\sigma_{\varepsilon}^{2}}$ |  |
| Concordance Correlation Coefficient (CCC) | S2 | $\frac{\sigma_{\alpha}^{2}+\sigma_{\alpha\gamma}^{2}}{\sigma_{\alpha}^{2}+\sigma_{\alpha\gamma}^{2}+\sigma_{\alpha\beta}^{2}+\beta_{\gamma}^{2}+\sigma_{\varepsilon}^{2}}$  $\beta_{\gamma}^{2}=\frac{1}{2p}\sum_{t=1}^{p} \left( \hat{u}_{1t}-\hat{u}_{2t} \right)^{2}-\frac{\sigma_{\alpha\beta}^{2}+\sigma_{e}^{2}}{n}$ | $\sigma_{\alpha}^{2}$: health facility variance  $\sigma_{\alpha\gamma}^{2}$: health facility:year variance  $\sigma_{\alpha\beta}^{2}$: health facility:data variance  $\sigma_{\varepsilon}^{2}$: residual variance  $\beta_{\gamma}^{2}$: data fixed effect, corrected for repeated measures  $p$: number of time points  $\hat{u}_{1t}-\hat{u}_{2t}$: fixed effect of time point t  $n$: number of facilities |

*Associations with Time and Facility Characteristics*

**Figure A.** Change in facility-level agreement ratios for 12 health facilities included in both 2017 and 2019 datasets. Similar to the full set of health facilities, changes in agreement ratios over time do not follow a consistent pattern; BC treated and PLHIV may show general improvement, with the exception of one health facility.

**Equation C.**

$$Y_{it}=\mu+\alpha_{i}+\gamma_{t}+\rho_{it}+e_{it}$$

$Y_{it}$= difference in log-transformed counts (surveillance data – research data)

$\mu$ = overall mean

$\alpha_{i}$ = random health facility effect

$\gamma_{t}$ = fixed year effect (*note that this term was only included in models for BC treated and Taking ART*)

$\rho_{it}$= fixed effect for one of the following: Health facility level (County vs. Subcounty [ref.]), Region (Eastern vs. Central [ref.]), or TB testing volume (number of smear examinations at facility *j* in year *i*)

$e_{it}$ = random error effect

**Table B.** Associations between health center characteristics and agreement between data sources (ratio).

|  | **Smear Positive** | **Xpert Positive** | **BC Treated** | **CD Treated** | **PLHIV** | **Taking ART** |
| --- | --- | --- | --- | --- | --- | --- |
|  | Coefficient  (p-value) | Coefficient  (p-value) | Coefficient  (p-value) | Coefficient  (p-value) | Coefficient  (p-value) | Coefficient  (p-value) |
| Health Center Level |  |  |  |  |  |  |
| *County* | 0.19  (0.43) | 0.079  (0.74) | -.052  (0.52) | -0.017  (0.88) | 0.013  (0.92) | 0.24  (0.11) |
| *Sub-county* | (ref.) | (ref.) | (ref.) | (ref.) | (ref.) | (ref.) |
| Region |  |  |  |  |  |  |
| *Eastern* | -0.27  (0.29) | 0.23  (0.37) | -0.10  (0.23) | -0.018  (0.89) | -0.11  (0.41) | -0.16  (0.32) |
| *Central* | (ref.) | (ref.) | (ref.) | (ref.) | (ref.) | (ref.) |
| TB testing volume | 0.0001  (0.19) | 4.8e-05  (0.95) | 0.0005 (0.09) | 5.5e-04 (0.18) | 0.0004  (0.26) | 7.199e-04  (0.15) |
